# A Cross-Sectional Study on Gluteal Muscles in Patients with Ankylosing Spondylitis at Different Stages of Hip Involvement

**DOI:** 10.3390/jcm12020464

**Published:** 2023-01-06

**Authors:** Tao Bian, Liang Zhang, Siliang Man, Hongchao Li, Weiyi Li, Yixin Zhou

**Affiliations:** 1 Department of Orthopedic Surgery, Beijing Jishuitan Hospital, Fourth Clinical College of Peking University, No. 31 Xinjiekou East Street, Xicheng District, Beijing 100035, China; 2 Department of Rheumatology and Immunology, Beijing Jishuitan Hospital, Fourth Clinical College of Peking University, No. 31 Xinjiekou East Street, Xicheng District, Beijing 100035, China; 3 Department of Rehabilitation, Beijing Jishuitan Hospital, Fourth Clinical College of Peking University, No. 31 Xinjiekou East Street, Xicheng District, Beijing 100035, China

**Keywords:** ankylosing spondylitis, gluteal muscle, hip involvement in ankylosing spondylitis, muscle cross-sectional area, muscle radiodensity

## Abstract

Hip involvement in ankylosing spondylitis (AS) is associated with severe functional impairment, and early diagnosis can improve the disease prognosis. We investigated gluteal muscle cross-sectional area (CSA) and radiodensity at different stages of hip involvement and their associations with AS-related clinical and laboratory parameters. This cross-sectional study included 83 patients with AS and 83 age- and sex-matched controls. Patients with AS were divided into three groups according to the Bath Ankylosing Spondylitis Radiology Hip Index system. The CSA and radiodensity of the gluteus maximus, medius, and minimus muscles were measured using computed tomography images. Muscle parameters were compared, and their relationships with clinical and laboratory parameters were evaluated. For the gluteus maximus, patients with AS had a lower CSA than controls, regardless of the degree of hip involvement. For the gluteus medius and minimus, patients with moderate/advanced hip involvement had significantly lower CSA and radiodensity than those with mild to no hip involvement. The severity of hip involvement was negatively associated with muscle parameters. CSA of the gluteus maximus decreased in early-stage hip involvement without any changes in radiographs, while radiodensity decreased in the later stages. Muscle parameters on computed tomography may be a more sensitive indicator than radiographic findings.

## 1. Introduction

Ankylosing spondylitis (AS) is a chronic inflammatory disease that commonly involves the axial spine and sacroiliac joints. Hip involvement occurs in 24–36% of patients with AS, causing decreased hip movement and impaired hip function and negatively affecting prognosis [1,2]. Hip involvement is usually diagnosed based on clinical symptoms and radiological examinations [1]. Radiography is the most widely used and validated method to evaluate the severity of hip involvement [2]. However, plain radiographs can only display untreatable structural changes in the late stage of hip involvement. Some studies have focused on computed tomography (CT) or magnetic resonance imaging (MRI) to reflect early changes in the hip joint [2,3,4].

Recently, several studies have paid more attention to pathological changes in the muscles of patients with AS; these changes may happen early in the disease progression and may be more sensitive for early diagnosis [5,6]. However, these studies have only focused on the paravertebral muscles rather than the gluteal muscles. The gluteal muscles are crucial in providing stability and mobility to the hips. Involvement of the gluteal muscles can result in pain, movement limitation, and poor pelvic support [7]. Moreover, studies focusing on gluteal muscle changes in patients with AS are limited in the literature. It is unknown whether the gluteal muscle is affected in the early stage of hip involvement in AS or not. Muscle degeneration usually manifests in two ways: a decrease in the size of the affected muscle and an increase in the number of fat deposits in the affected muscle [6]. Measurement of muscle cross-sectional area (CSA) and radiodensity in CT images is a reliable and validated quantitative method to reflect muscle size and quality, respectively [8,9,10,11].

This study aimed to investigate the changes in gluteal muscle CSA and radiodensity in patients with AS at different stages of hip involvement and to find their associations with AS-related clinical and laboratory parameters. We hypothesized that muscle parameters might change during the early stage of hip involvement in AS and, therefore, might serve as a sensitive indicator for early hip involvement.

## 2. Materials and Methods

### 2.1. Study Participants

This was a single-center cross-sectional study. The inclusion criteria were as follows: a diagnosis of AS according to the modified New York criteria [12]; age between 16 and 55 years. The exclusion criteria were as follows: congenital or childhood hip diseases; a history of infection, trauma, tumor, or surgery in the hip joints; inflammatory arthritis and connective tissue diseases other than AS; and the use of corticosteroids or biological agents. From October 2017 to June 2021, 83 patients were included in the study.

In addition, 83 age- and sex-matched healthy control subjects were selected from the institutional radiology database. The control subjects had undergone CT scans for other reasons and had no previous hip pain, surgery, dysplasia, fracture, or limited range of motion.

### 2.2. Demographic Data and Clinical Parameters

Demographic data, including sex and body mass index (BMI) and AS-related clinical information (including age at disease onset, age at outpatient visit, disease duration, diagnosis delay, family history, and medication status) were collected in the outpatient setting using questionnaires. The use of nonsteroidal anti-inflammatory drugs (NSAIDs) and conventional synthetic disease-modifying antirheumatic drugs (csDMARDs) was recorded. Disease activity and functional status were evaluated using the Bath Ankylosing Spondylitis Disease Activity Index (BASDAI) and the Bath Ankylosing Spondylitis Functional Index (BASFI) [13], respectively. Patient quality of life was evaluated using the Short Form-12 (SF-12) [14] and Ankylosing Spondylitis Quality of Life (ASQoL) scales [13]. Hip joint function was evaluated using the Harris hip score (HHS) by orthopedic surgeons [15].

Laboratory data at the initial visit (including serum erythrocyte sedimentation rate; serum C-reactive protein [CRP], albumin, and hemoglobin levels; and human leukocyte antigen B27 [HLA-B27] status) were collected from patient medical records.

### 2.3. Radiographic Data Collection

Baseline anteroposterior radiographs of both hips were collected at the outpatient visits. The severity of hip involvement was graded using the Bath Ankylosing Spondylitis Radiology Hip Index (BASRI-hip) system on a scale of 0–4 (0: normal, no change; 1: suspicious, possible focal joint space narrowing; 2: minimal, definite narrowing, leaving a circumferential joint space of >2 mm; 3: moderate, narrowing, but with circumferential joint space narrowing of ≤2 mm or bone-on-bone apposition of <2 cm; 4: severe, bone deformity or bone-on-bone apposition of >2 cm, or total hip arthroplasty [THA]) [16]. All patients with AS were divided into three groups based on their BASRI-hip grades as follows: group 1, no radiographic hip involvement (BASRI-hip score of ≤1); group 2, mild radiographic hip involvement (BASRI-hip score of 2); and group 3, moderate to advanced radiographic hip involvement (BASRI-hip score of ≥3) [16].

### 2.4. Muscle Parameter Measurement

CT images of the hip were obtained from patients in the supine position using the Toshiba Aquilion CT scanner (Toshiba Medical Systems Division, Tokyo, Japan). The scan parameters were as follows: 120 kVp, 125 mAs, 50 cm field of view, 512 × 512 matrix, and a 1 mm-thick reconstructed slice thickness.

SliceOmatic software (version 5.0, TomoVision, Magog, Quebec, Canada) was used for image analysis. Muscle CSA and radiodensity (mean Hounsfield Units [HU]) were measured on one slice each. The gluteus maximus muscle was measured just above the femoral head [17,18], and the gluteus medius and minimus muscles, together, were measured at the inferior point of the sacroiliac joint [18,19]. Free-hand painting of the gluteal muscles was performed using the region growing mode of the software, which selects tissue within the preset HU intensity thresholds (−30 to 150 HU) [10,20]. Muscle CSA and radiodensity values were reported automatically by the software. Measurements were recorded twice by a single assessor at an interval of four weeks, and the mean values were used for analyses [19]. The skeletal muscle index (SMI), calculated by dividing the muscle CSA (cm^2^) by the patient’s height squared (m^2^), was also calculated [21,22].

### 2.5. Ethics

This study was approved by the Beijing Jishuitan Hospital Institutional Review Board (approval number 202104-18) and has been performed in accordance with the ethical standards of the 1964 Declaration of Helsinki and its later amendments. All participants provided written informed consent.

### 2.6. Statistical Analysis

The normality of continuous data was evaluated using the Shapiro–Wilk test. Normally distributed data are reported as means ± standard deviations and compared using one-way analyses of variance (ANOVA, including post hoc analysis). Non-normally distributed data are reported as medians (interquartile ranges) and compared using Kruskal–Wallis test. Categorical data are reported as counts and percentages and compared using Pearson’s Chi-square or Fisher’s exact test. Spearman’s correlation coefficient was used to evaluate the relationships between muscle- and clinical-related parameters. Linear regression analyses were performed to assess the relationship between muscle parameters (dependent variables) and the severity of hip involvement while controlling for sex, age, and BMI. Test-retest reliability was calculated using the intraclass correlation coefficient (ICC). All statistical analyses were performed using SPSS software (version 20.0; IBM Corp., Armonk, NY, USA), and significance was set at a two-tailed *p*-value of <0.05.

## 3. Results

### 3.1. Characteristics of Patients with AS

The demographic, clinical, and laboratory data of patients with AS are presented in Table 1 and Table 2.

Among the 83 patients with AS, 72 (86.7%) were men, and 11 (13.3%) were women. Using the BASRI-hip grade, 34 (41.0%) patients were determined to have no radiographic hip involvement (BASRI-hip score of ≤1, group 1); 24 (28.9%), mild (BASRI-hip score of 2, group 2); and 25 (30.1%), moderate to advanced (BASRI-hip score of ≥3, group 3). Compared with those in groups 1 and 2, patients in group 3 experienced disease onset at a significantly earlier age (median 20.0 years; group 1, 24.5 years, *p* = 0.001; group 2, 24.5 years, *p* = 0.027). Patients in group 3 had a significantly longer disease duration than those in group 1 (median 13.0 years vs. 6.0 years, respectively; *p* = 0.014). No differences in sex, age at outpatient visit, diagnosis delay, BMI, percentage of family history, NSAID and csDMARD use, or HLA-B27 positivity were noted among the three groups. Compared with those in group 1, patients in group 3 had elevated CRP levels (*p* = 0.039); no differences in other laboratory data were found. Patients in group 3 had worse clinical scores (BASFI, ASQOL, and SF-12 PCS) than those in group 1 and had a significantly lower HHS than those in the other groups (median 72.5; group 1, 84.5; group 2, 85.0; *p* < 0.001).

### 3.2. Comparison of Muscle Parameters between Patients with AS and Controls

The measurements had good test-retest reliability, with ICC values ranging from 0.968–0.990. Comparisons of muscle CSA, radiodensity, and SMI between patients with AS and controls (including pairwise comparisons) are presented in Table 3.

For the gluteus maximus muscle, compared with the control group, patients with AS had a lower CSA—regardless of the degree of hip involvement (median 45.05 cm^2^; group 1, 40.68 cm^2^, *p* = 0.013; group 2, 36.72 cm^2^, *p* < 0.001; group 3, 32.54 cm^2^, *p* < 0.001). Patients in group 1 had a higher CSA than the patients in group 3 (*p* = 0.002). On the other hand, no difference in radiodensity between patients in group 1 and the control group was found (control group, 39.90 HU; group 1, 40.17 HU, *p* = 1.000). Other differences in radiodensity were similar to the trends seen in CSA values.

For the gluteus medius and minimus muscles, patients in group 3 had significantly lower CSA (*p* < 0.001) and radiodensity (group 1, *p* < 0.001, group 2, *p* = 0.034) values than those in the other groups. No significant differences in other comparisons were observed. The results of gluteal muscle SMI were similar to those of the trends seen in CSA values.

### 3.3. Association between Clinical and Muscle Parameters

The results of Spearman correlation analyses are presented in Table 4.

Male sex was correlated with a higher CSA of the gluteus medius and minimus muscles (r = 0.404, *p* < 0.001). Age at the outpatient visit was positively correlated with muscle CSA (gluteus maximus, r = 0.202, *p* < 0.01; gluteus medius and minimus, r = 0.219, *p* < 0.01) and negatively associated with radiodensity (gluteus maximus, r = −0.157, *p* < 0.05; gluteus medius and minimus, r = −0.172, *p* < 0.05). Age at disease onset was positively related to muscle CSA and radiodensity (*p* < 0.01). A longer disease duration was correlated with lower muscle radiodensity (*p* < 0.001). BMI was positively correlated with muscle CSA and SMI (r > 0.4; *p* < 0.001). A higher gluteus medius and minimus muscle radiodensity were associated with better clinical parameters (BASDAI, r = −0.178, *p* < 0.05; BASFI, r = −0.277, *p* < 0.001; ASQOL, r = −0.224, *p* < 0.01; SF-12 PCS, r = 0.329, *p* < 0.001). Muscle parameters were positively related to HHS and negatively related to radiography grade (*p* < 0.01).

### 3.4. Relationship between the Severity of Hip Involvement and Muscle Parameters

The results of multiple linear regression analyses are shown in Table 5.

The severity of hip involvement had a statistically significant inverse correlation with muscle parameters after controlling for sex, age, and BMI (standardized beta coefficient ranged from −0.390 to −0.128, *p* <0.01).

## 4. Discussion

In this study, we used CT images to evaluate the CSA and radiodensity of gluteal muscles in patients with AS at different stages of hip involvement. The most important finding was that the CSA of the gluteus maximus muscle decreased in the early stages of hip involvement when changes in radiographs were either absent or suspicious. Radiodensity of the gluteus maximus muscle decreased later than that of CSA. On the other hand, the CSA and radiodensity of the gluteus medius and minimus muscles were reduced in the late stage of hip involvement. Muscle quantity and quality were significantly reduced in the late stage of hip involvement and were negatively associated with the severity of hip involvement after controlling for sex, age, and BMI.

AS is a systemic inflammatory disease that usually affects the sacroiliac joint and vertebral column in its early stages. Previous studies have focused more on changes in the spinal column and have found that patients with AS have higher atrophy and fatty degeneration in paraspinal muscles, where this atrophy may be due to chronic inflammation, the effects of cytokines, and limitations in spinal mobility [5,23]. Besides, skeletal muscle fatty infiltration may be a contributing factor to sarcopenia, which is characterized by generalized loss of muscle mass and function [24]. Patients with AS had lower muscle mass and strength and a higher prevalence of sarcopenia [25]. Muscle CSA and radiodensity of the hip joint are correlated to those of the psoas muscles at the L3 level, where the psoas muscles may be used as a supplemental reference to screen for sarcopenia status [26]. Therefore, more information may be obtained from hip CT images to reflect whole-body composition statuses and thus guide early intervention. However, studies focusing on changes in gluteal muscle size and quality in AS patients are sparse.

To the best of our knowledge, this is the first study that focused on the gluteal muscles in patients with AS [5,6]. The gluteal muscles are crucial in maintaining proper hip joint function and stability and the positioning of the pelvis [27]. Degeneration of the muscle was reflected by decreased muscle mass or muscle CSA, lowered muscle density, and fatty infiltration, all of which can be measured from CT images without additional examination [6,28]. CT images can reflect not only the details of bone changes but also the degeneration in muscle tissues. Intramuscular fatty infiltration did not decrease muscle CSA but did lower the quality of the muscle, affecting its force-generating capacity [29]. Measuring radiodensity from CT images is a reliable and validated method to assess fatty infiltration in muscle tissues, wherein a 0.75–1 HU reduction in radiodensity corresponds to a 1% increase in adiposity [9,10]. In this study, we used the region growing function in the SliceOmatic software and selected the tissue with a radiodensity between −30 and 150 HU (the range of skeletal muscle) [10,20]. This method excluded intermuscular fatty infiltration and reflected actual skeletal muscle area and intramuscular fatty infiltration. Compared with muscle strength measurement, muscle CSA and radiodensity measurement is a more objective and sensitive method to reflect individual muscle characteristics, and these values correlate with muscle strength and physical performance [11,17,20,29,30].

In our study, we found that patients with AS with moderate or advanced hip involvement had significantly lower gluteal muscle CSA and radiodensity than those with no hip involvement—a finding that is similar to those of previous studies [5,31]. In patients with advanced osteoarthritis (OA), the quantity and quality of hip muscles significantly decrease, and muscle degeneration correlates with poorer outcomes after THA [27,30,31,32,33]. Muscle degeneration in patients with AS may be caused by chronic inflammation, cytokine release, and reduced mobility in the late stages of AS [33].

Our study is valuable in showing that the CSA of the gluteus maximus muscle decreased in the early stage of hip involvement in AS, indicating that it may be a sensitive indicator of hip involvement before structural changes can be observed on radiographs. The gluteus maximus muscle is vital in the gait cycle and many other daily activities [31]. In addition, the gluteus maximus muscle CSA is positively correlated with proximal femur volumetric bone mineral density [34,35]. Therefore, patients with muscle changes in the early stage of hip involvement may be detected by CT images and benefit from early physical rehabilitation therapy and muscle strengthening exercises to halt further muscle atrophy and bone loss. Regarding gluteus medius and minimus muscles, we found that the CSA decreased in the late stage of hip involvement in AS, consistent with the findings of a previous study [36]. Grimaldi et al. reported that the gluteus medius and minimus muscles atrophied significantly in advanced hip OA; however, muscle size did not decrease in the early stages of the pathology [36]. The degeneration difference between the gluteus maximus and the gluteus medius and minimus muscles may be due to the differences in the anatomy, insertion, innervation, and function of the two muscle groups [33]. Rehabilitation exercises targeting specific muscle groups can achieve better results and improve the patient’s quality of life.

Wang et al. reported that muscle density was a better parameter to reflect muscle strength and physical performance than muscle mass and size [20]. However, we found that the radiodensity of the gluteal muscles showed no difference in the early stage of hip involvement in AS and decreased in the late stage. This could be explained by the similar HHS between groups 1 and 2, indicating no apparent dysfunction in patients with mild radiographic hip involvement included in our study. We found that the radiodensity of the gluteal muscles was negatively associated with disease duration, similar to the findings of a previous study [5]. In addition, only the radiodensity of the gluteus medius and minimus muscles were correlated with the clinical parameters. Muscle CSA was not associated with disease duration or clinical parameters. Our results were similar to those of Kim et al., who found that skeletal muscle mass was not associated with quality of life in patients with AS [37]. However, their patients had relatively short disease durations (mean six years) and had similar muscle mass compared to the general population. In our study, CSA and radiodensity were positively associated with HHS, with the radiodensity of the gluteus medius and minimus muscles having the highest correlation coefficient. Physical therapy focusing on gluteal muscles in the early stage of hip involvement in AS may prevent further reduction in muscle quality and hip joint function.

Together, our study showed that the gluteal muscles degenerated in the early stage of hip involvement in AS, which has many implications for clinical practice and future research. For example, gluteal muscle degeneration can become a useful marker for the early detection of hip involvement, and thus patients may benefit from early intervention to prevent further muscle degeneration. Furthermore, muscle parameters of the hip joint can also reflect whole-body composition status. Future studies should investigate on other muscles around the hip joints and their changes after intervention. This study has some limitations. First, this was a cross-sectional study with a relatively small number of participants, which may have selection bias and prevent determining causal relationships. Therefore, further longitudinal studies are needed to investigate causality. Second, the CT images of the hip did not include the whole range of the gluteus maximus muscle, thus preventing the measurement of the muscle volume. However, previous studies found that the CSA at the specific site strongly correlated with muscle volume and strength and we selected the measurement site accordingly [17,18,19]. Although MRI is a better method to reflect soft tissue, we used CT in this study because CT can also quantitatively measure the radiodensity of muscles. Muscle CSA and radiodensity can quantitatively reflect intermuscular and intramuscular fatty infiltration. CT images are more convenient to obtain and can be better generalized.

## 5. Conclusions

We found that gluteal muscle CSA and radiodensity are correlated with the severity of hip involvement. The CSA of the gluteus maximus muscle decreased in the early stage of hip involvement in AS. Our study found that muscle parameters on CT images may be a more sensitive indicator than radiographic findings of disease progression and early hip involvement. Further studies should investigate this in other muscles around the hip joints and their changes after intervention. Patients with evidence of early hip involvement may benefit from early intervention, including physical rehabilitation therapy and pharmacological treatment, thus preventing functional impairment, and muscle parameters can be used as an indicator of the effect of the intervention.

## Figures and Tables

**Table 1:** Comparison of demographic data among patients with ankylosing spondylitis, based on the degree of hip involvement.

	Total Patients with AS (*n* = 83)	Group 1: BASRI-Hip ≤ 1 (*n* = 34)	Group 2: BASRI-Hip = 2 (*n* = 24)	Group 3: BASRI-Hip ≥ 3 (*n* = 25)	*p* Value	Adjusted *p* Value (Group 1 vs. 2)	Adjusted *p* Value (Group 1 vs. 3)	Adjusted *p* Value (Group 2 vs. 3)
Male sex, *n* (%)	72 (86.7%)	29 (85.3%)	21 (87.5%)	22 (88.0%)	1.000			
Age at outpatient visit (years)	33.0 (28.0–37.0)	33.5 (29.0–37.5)	32.0 (28.3–37.0)	33.0 (25.0–39.5)	0.956			
Age at onset (years)	23.0 (19.0–28.0)	24.5 (22.0–30.0)	24.5 (19.0–28.0)	20.0 (15.0–23.5)	0.001	1.000	0.001	0.027
Disease duration (years)	8.0 (2.0–13.0)	6.0 (1.8–10.3)	7.0 (4.3–9.8)	13.0 (2.5–20.5)	0.017	1.000	0.014	0.242
Diagnosis delay (years)	3.0 (1.0–7.0)	2.0 (0.8–5.5)	3.5 (0–5.0)	4.0 (1.5–15.0)	0.132			
BMI (kg/m^2^)	24.5 ± 4.3	24.9 ± 3.7	24.9 ± 4.6	23.7 ± 4.7	0.506			
Family history, *n* (%)	28 (33.7%)	15 (44.1%)	5 (20.8%)	8 (32.0%)	0.174			
Use of NSAIDs, *n* (%)	58 (69.9%)	25 (73.5%)	15 (62.5%)	18 (72.0%)	0.625			
Use of csDMARDs, *n* (%)	44 (53.0%)	20 (58.8%)	13 (54.2%)	11 (44.0%)	0.527			

Data are presented as means ± standard deviations, median (interquartile range), or as frequencies and percentages. AS, ankylosing spondylitis; BASRI-hip, Bath Ankylosing Spondylitis Radiology Hip Index; BMI, body mass index; NSAIDs, non-steroidal anti-inflammatory drugs; csDMARDs, conventional synthetic disease-modifying anti-rheumatic drugs.

**Table 2:** Comparison of clinical and laboratory data among patients with ankylosing spondylitis, based on the degree of hip involvement.

	Total Patients with AS (*n* = 83)	Group 1: BASRI-Hip ≤ 1 (*n* = 34)	Group 2: BASRI-Hip = 2 (*n* = 24)	Group 3: BASRI-Hip ≥ 3 (*n* = 25)	*p* Value	Adjusted *p* Value (Group 1 vs. 2)	Adjusted *p* Value (Group 1 vs. 3)	Adjusted *p* Value (Group 2 vs. 3)
BASDAI	3.8 (1.9–5.5)	3.4 (1.8–5.4)	3.0 (1.4–4.8)	4.9 (3.0–6.6)	0.026	1.000	0.068	0.045
BASFI	3.2 (1.2–6.5)	2.0 (1.0–3.8)	2.8 (1.1–6.4)	6.2 (2.2–7.4)	0.008	1.000	0.007	0.103
SF-12 PCS	38.5 (29.8–48.4)	48.2 (34.4–52.9)	40.2 (30.0–48.0)	33.5 (24.1–39.6)	0.011	0.384	0.008	0.496
SF-12 MCS	46.0 (33.2–53.6)	47.7 (34.7–54.3)	46.8 (29.0–53.6)	42.8 (33.2–50.8)	0.729			
ASQOL	5.5 (1.0–8.0)	3.0 (0–7.0)	6.0 (0.3–8.0)	7.0 (5.0–10.0)	0.032	0.908	0.026	0.425
HHS	82.0 (74.8–90.0)	84.5 (81.0–90.0)	85.0 (78.3–91.5)	72.5 (65.0–78.0)	<0.001	1.000	<0.001	<0.001
HLA-B27 positivity, *n* (%)	75 (90.4%)	29 (85.3%)	24 (100%)	22 (88.0%)	0.161			
ESR (mm/h)	25.0 (13.0–40.0)	18.0 (12.0–30.0)	29.0 (13.0–42.0)	29.0 (21.5–42.5)	0.119			
CRP (mg/L)	13.6 (7.2–34.1)	8.4 (4.7–25.7)	15.7 (9.4–28.0)	23.9 (8.6–47.7)	0.033	0.202	0.039	1.000
ALB (g/L)	44.9 (42.4–47.1)	44.9 (43.2–47.7)	46.0 (43.9–46.8)	44.0 (40.0–47.0)	0.324			
HGB (g/L)	141.0 (132.0–150.8)	145.0 (132.0–153.0)	138.0 (128.3–146.8)	144.0 (128.5–150.0)	0.451			

Data are presented as median (interquartile range) or as frequencies and percentages. AS, ankylosing spondylitis; BASRI-hip, Bath Ankylosing Spondylitis Radiology Hip Index; BASDAI, Bath ankylosing spondylitis disease activity index; BASFI, Bath ankylosing spondylitis functional index; SF-12 PCS, short form-12 physical component summary; SF-12 MCS, short form-12 mental component summary; ASQOL, ankylosing spondylitis quality of life; HHS, Harris hip score; HLA-B27, human leucocyte antigen-B27; ESR, erythrocyte sedimentation rate; CRP, C-reactive protein; ALB, albumin; HGB, hemoglobin.

**Table 3:** Comparison of muscle parameters between patients with ankylosing spondylitis versus controls.

Parameters	Control Group (*n* = 83)	Group 1: BASRI-Hip ≤ 1 (*n* = 34)	Group 2: BASRI-Hip = 2 (*n* = 24)	Group 3: BASRI-Hip ≥ 3 (*n* = 25)	*p* Value	Adjusted *p* Value for Groups					
						0 vs. 1	0 vs. 2	0 vs. 3	1 vs. 2	1 vs. 3	2 vs. 3
G.MaxM CSA (cm^2^)	45.05 (38.32–52.91)	40.68 (32.55–48.21)	36.72 (31.02–45.79)	32.54 (22.84–40.51)	<0.001	0.013	<0.001	<0.001	0.786	0.002	0.324
G.MaxM radiodensity (HU)	39.90 (34.16–43.96)	40.17 (34.14–43.91)	35.59 (30.32–40.05)	32.93 (26.87–41.31)	<0.001	1.000	0.016	0.001	0.126	0.014	1.000
G.MaxM SMI (cm^2^/m^2^)	15.18 (13.15–17.32)	13.97 (11.59–16.58)	12.29 (10.56–15.96)	11.27 (7.75–13.29)	<0.001	0.047	<0.001	<0.001	0.646	0.001	0.338
G.Med/MinM CSA (cm^2^)	44.46 (39.99–48.69)	43.48 (38.13–47.88)	45.16 (39.90–52.04)	37.24 (31.01–42.23)	<0.001	1.000	1.000	<0.001	1.000	<0.001	<0.001
G.Med/MinM radiodensity (HU)	48.43 (43.73–51.50)	49.75 (44.15–51.53)	46.29 (42.93–48.29)	40.63 (29.27–46.35)	<0.001	1.000	0.138	<0.001	0.106	<0.001	0.034
G.Med/MinM SMI (cm^2^/m^2^)	14.82 (13.47–16.13)	14.84 (12.97–16.04)	15.51 (13.25–17.68)	12.63 (10.40–14.18)	<0.001	1.000	0.736	<0.001	0.913	<0.001	<0.001

Data are presented as median (interquartile range). BASRI-hip, Bath Ankylosing Spondylitis Radiology Hip Index; G.MaxM, gluteus maximus muscle; CSA, cross-sectional area; HU, Hounsfield units; SMI, skeletal muscle index; G.Med/MinM, gluteus medius, and minimus muscle.

**Table 4:** Spearman correlation coefficients between clinical data and muscle parameters.

Correlation Coefficients (rs)	G.MaxM CSA	G.MaxM Radiodensity	G.MaxM SMI	G.Med/MinM CSA	G.Med/MinM Radiodensity	G.Med/MinM SMI
Sex	0.117	0.133	−0.056	0.404 ^c^	0.118	0.219 ^b^
Age at outpatient visit (years)	0.202 ^b^	−0.157 ^a^	0.242 ^b^	0.219 ^b^	−0.172 ^a^	0.270 ^c^
Age at onset (years)	0.226 ^b^	0.075	0.236 ^b^	0.239 ^b^	0.212 ^b^	0.233 ^b^
Disease duration (years)	0.023	−0.274 ^c^	0.037	0.105	−0.354 ^c^	0.149
Diagnosis delay (years)	−0.068	−0.13	−0.089	0.070	−0.192 ^a^	0.067
Body mass index (kg/m^2^)	0.470 ^c^	−0.128	0.478 ^c^	0.432 ^c^	0.034	0.498 ^c^
Family history	−0.030	0.087	−0.025	−0.011	0.121	−0.013
Use of non-steroidal anti-inflammatory drugs	−0.132	−0.094	−0.079	−0.121	−0.025	−0.055
Use of csDMARDs	−0.047	0.042	−0.013	−0.092	0.131	−0.035
Human leucocyte antigen-B27 positivity	−0.129	0.032	−0.118	0.012	0.059	0.084
Erythrocyte sedimentation rate (mm)	−0.192 ^a^	−0.152	−0.150	−0.255 ^b^	−0.268 ^b^	−0.169 ^a^
C-reactive protein (mg/L)	−0.191 ^a^	−0.113	−0.198 ^a^	−0.128	−0.182 ^a^	−0.101
Albumin (g/L)	0.185 ^a^	0.099	0.083	0.233 ^b^	0.204 ^a^	0.133
Hemoglobin (g/L)	0.251 ^b^	0.105	0.153	0.270 ^b^	0.128	0.152
BASDAI	−0.123	−0.103	−0.049	−0.166 ^a^	−0.178 ^a^	−0.089
Bath ankylosing spondylitis functional index	−0.056	−0.144	−0.033	−0.177 ^a^	−0.277 ^c^	−0.132
Ankylosing spondylitis quality of life	−0.012	−0.098	0.048	−0.070	−0.224 ^b^	−0.009
SF-12 physical component summary	0.006	0.124	−0.036	0.108	0.329 ^c^	0.042
SF-12 mental component summary	0.045	−0.129	0.022	0.064	−0.103	0.042
Harris hip score	0.243 ^b^	0.227 ^b^	0.249 ^b^	0.262 ^b^	0.402 ^c^	0.270 ^c^
BASRI-hip grade	−0.300 ^c^	−0.248 ^b^	−0.305 ^c^	−0.278 ^c^	−0.423 ^c^	−0.258 ^b^

^a^ *p* < 0.05; ^b^ *p* < 0.01; ^c^ *p* < 0.001. G.MaxM, gluteus maximus muscle; CSA, cross-sectional area; SMI, skeletal muscle index; G.Med/MinM, gluteus medius and minimus muscle; csDMARDs, conventional synthetic disease modifying anti-rheumatic drugs; BASDAI, Bath ankylosing spondylitis disease activity index; SF-12, short form-12; BASRI-hip, Bath Ankylosing Spondylitis Radiology Hip Index.

**Table 5:** The relationship between the severity of hip involvement and muscle parameters, after controlling for sex, age, and BMI.

Dependent Variables	R^2^	Adjusted R^2^	Independent Variables	Beta Coefficient	Standardized Beta coefficient	*p* Value	95% CI	
G.MaxM CSA	0.376	0.368	Sex	6.644	0.192	<0.001	3.628	9.659
			Age	0.001	0.001	0.993	−0.118	0.119
			BMI	1.037	0.372	<0.001	0.790	1.285
			Group	−4.153	−0.390	<0.001	−5.078	−3.229
G.MaxM density	0.196	0.187	Sex	3.692	0.149	0.003	1.250	6.134
			Age	−0.241	−0.253	<0.001	−0.337	−0.145
			BMI	−0.205	−0.103	0.044	−0.406	−0.005
			Group	−2.253	−0.297	<0.001	−3.001	−1.504
G.MaxM SMI	0.338	0.330	Sex	0.433	0.038	0.400	−0.577	1.443
			Age	0.017	0.040	0.393	−0.022	0.057
			BMI	0.356	0.392	<0.001	0.273	0.439
			Group	−1.264	−0.365	<0.001	−1.574	−0.954
G.Med/MinM CSA	0.481	0.475	Sex	13.281	0.499	<0.001	11.171	15.390
			Age	0.134	0.131	0.002	0.052	0.217
			BMI	0.806	0.377	<0.001	0.633	0.979
			Group	−1.509	−0.185	<0.001	−2.156	−0.862
G.Med/MinM density	0.207	0.198	Sex	2.362	0.103	0.040	0.107	4.618
			Age	−0.187	−0.211	<0.001	−0.276	−0.099
			BMI	0.120	0.065	0.204	−0.065	0.305
			Group	−2.637	−0.373	<0.001	−3.329	−1.946
G.Med/MinM SMI	0.374	0.366	Sex	2.802	0.330	<0.001	2.065	3.539
			Age	0.062	0.188	<0.001	0.033	0.090
			BMI	0.285	0.418	<0.001	0.225	0.346
			Group	−0.333	−0.128	0.004	−0.559	−0.107

G.MaxM, gluteus maximus muscle; CSA, cross-sectional area; SMI, skeletal muscle index; G.Med/MinM, gluteus medius, and minimus muscle; BMI, body mass index.

## Data Availability

The datasets used and/or analyzed during the current study are available from the corresponding author upon reasonable request.
